# Multiplexed Immunohistochemistry and Digital Pathology as the Foundation for Next-Generation Pathology in Melanoma: Methodological Comparison and Future Clinical Applications

**DOI:** 10.3389/fonc.2021.636681

**Published:** 2021-03-29

**Authors:** Yannick Van Herck, Asier Antoranz, Madhavi Dipak Andhari, Giorgia Milli, Oliver Bechter, Frederik De Smet, Francesca Maria Bosisio

**Affiliations:** ^1^Department of Oncology, KU Leuven, Leuven, Belgium; ^2^Laboratory for Translational Cell and Tissue Research, Department of Imaging and Pathology, KU Leuven, Leuven, Belgium; ^3^Laboratory for Precision Cancer Medicine, Translational Cell and Tissue Research Unit, Department of Imaging and Pathology, KU Leuven, Leuven, Belgium

**Keywords:** melanoma, multiplex, single cell, digital pathology, spatial proteomics

## Abstract

The state-of-the-art for melanoma treatment has recently witnessed an enormous revolution, evolving from a chemotherapeutic, “one-drug-for-all” approach, to a tailored molecular- and immunological-based approach with the potential to make personalized therapy a reality. Nevertheless, methods still have to improve a lot before these can reliably characterize all the tumoral features that make each patient unique. While the clinical introduction of next-generation sequencing has made it possible to match mutational profiles to specific targeted therapies, improving response rates to immunotherapy will similarly require a deep understanding of the immune microenvironment and the specific contribution of each component in a patient-specific way. Recent advancements in artificial intelligence and single-cell profiling of resected tumor samples are paving the way for this challenging task. In this review, we provide an overview of the state-of-the-art in artificial intelligence and multiplexed immunohistochemistry in pathology, and how these bear the potential to improve diagnostics and therapy matching in melanoma. A major asset of in-situ single-cell profiling methods is that these preserve the spatial distribution of the cells in the tissue, allowing researchers to not only determine the cellular composition of the tumoral microenvironment, but also study tissue sociology, making inferences about specific cell-cell interactions and visualizing distinctive cellular architectures - all features that have an impact on anti-tumoral response rates. Despite the many advantages, the introduction of these approaches requires the digitization of tissue slides and the development of standardized analysis pipelines which pose substantial challenges that need to be addressed before these can enter clinical routine.

## Introduction

### Next-Generation Pathology and Personalized Medicine in Melanoma

The oncological treatment of melanoma has radically changed over the past 10 years: it evolved from a “one-fits-all” chemotherapeutic treatment with DTIC (1) to a more tailored setting where therapies are only given when patient- and tumor-specific features are present. This evolution toward personalized therapy was ignited by the observation that specific drugs were only clinically effective in the presence of a specific mutation (2–10). In addition, following the first successes with IL-2 therapy (11–13), immunotherapy was re-evaluated leading to the identification and implementation of checkpoint inhibitor therapy, a type of immunotherapy based on blocking the breaks that normally prevent the immune system from becoming hyperactivated (14–17). While oncology is gradually moving toward personalized treatments, also pathological assessments need to progress to cope with the need for in-depth characterizations of tumor tissues from individual patients. Salto-Tellez et al. have previously discussed how pathology, a discipline originally based on the evaluation of tissue morphology by hematoxylin-eosin (HE) staining, witnessed 3 main revolutions: first, the introduction of immunohistochemistry (IHC) in the 80s; second, the adoption of molecular techniques in pathology (molecular pathology, MP; mostly next-gen sequencing); and, most recently, the development of artificial intelligence (AI) tools to support the pathologist to evaluate and interpret the different features (18). While tools from the first two revolutions are nowadays fully embedded in routine clinical work and represent the earliest steps toward personalized medicine, the third revolution is still awaiting its breakthrough.

From the available tools, MP is the most advanced as it reached the required level of specificity to represent the state-of-the-art. It is mostly based on next-generation sequencing through which it allows the identification of genetic aberrations, either by analyzing focused gene panels or whole genome sequencing. In melanoma, the mutational profile is nowadays used to support diagnostics but also to select the most appropriate treatment. For the former, the new WHO Skin Cancer classification has identified 9 molecular pathways in which the melanocytic lesions can be classified based on the type and number of genetic alterations involved (19). Each of these pathways is further divided in 3 categories with different biological behavior (benign, intermediate and malignant) that can also be predicted according to the number of genetic alterations (≤1, 2 and >2 respectively) (19). The choice of treatment, on the other hand, is primarily based on the presence of targetable mutations, such as BRAF V600 mutations, for which specific therapies are available (2–10).

While NGS methods are constantly improving and evolving, the use of IHC hardly changed over the past 20 years. Indeed, as opposed to NGS analyses that typically cover 10-100 genes simultaneously, conventional IHC allows to stain tissue sections one marker at the time. As such, the analysis of multiple biomarkers typically requires the analysis of serial sections which may be a limiting step in small biopsies where only small amounts of materials are available. Moreover, by its inability to investigate the co-expression of several markers in the same cell, important information is systematically missed. A workaround has been to analyze marker expression patterns in serial sections, but this approach does not achieve sufficient detail to get to a robust interpretation. As a consequence, conventional IHC has become largely insufficient to cope with the required level and depth by which tumor tissues for each individual patients should be analyzed. A striking example involves the use of PD-L1 as a single-plex marker for the prediction of immunotherapy response: even though it has been implemented in routine pathological assessments, its detection suffers from significant technical hurdles making it largely insufficient as a good predictive marker. Moreover, recent research suggests that the cell types that express PD-L1 and their location in the tissue is also of major importance. However, gaining such insights cannot be addressed by old pathological practices where a semi-quantitative eye-balling interpretation of the staining is used for subjective evaluation, and therefore requires the implementation of single cell-technologies that preserve the spatial distribution of the various cell types and their original state (20). Multiplexed IHC, a technological approach that harbors the potential to collect exactly this type of data, has witnessed major progress over the past 2-3 years, but still requires several adaptations. For instance, it relies on full image digitalization and extended computational analysis, a limitation (but also opportunity) that multiplexed IHC and artificial intelligence (AI) have in common for their further implementation in a clinical setting.

Even though digital pathology-based AI tools have already been developed and have shown some diagnostic, prognostic, and predictive potential comparable to standard molecular and genomic-based tests, digital pathology (i.e. the process of digitizing whole-slide images using advanced slide-scanning techniques) has not yet been introduced in hospitals at large scale. Recent advancements in multiplexed IHC anticipate an even more important role for AI in pathology. The plethora of data generated by multiplexed IHC where tens to hundreds of markers are measured in thousands to millions of cells in their spatial context, provides the ideal setting to exploit AI and deep learning methods in particular. One of the strongest aspects of deep learning is to discover hidden features (and their combinations) otherwise invisible by purely visual inspection, and correlate them with clinical data. The parallel advancement of multiplexed IHC and AI-based computational models represent an unprecedented scenario for the introduction of next-generation pathology in clinical practice, characterized by the more widespread usage of digital images and the introduction of artificial intelligence and deep learning tools on histopathological images.

In this review we discuss the state-of-the-art, the potential and the challenges linked to the introduction of next-generation pathology to the clinical practice of melanoma patients. All the studies considered in this review are summarized in **Table 1**.

**Table 1:** Overview of recent studies using digital pathology in melanoma. All studies are ordered according to time of publication.

Study	Main Objective	Study population	Method(s)	Main finding(s)/results
Makhzami et al. 2012 (21)	Improve the cell-type purity by performing laser-microdissection and investigate tissue-based transcriptomic data	Transgenic mice	IHC-guided laser microdissection	Optimized workflow of laser microdissection & stronger expression of five genes (M-MITF, TYR, STAT3, CCND1 and PAX3) in primary than metastatic melanoma
Bifulco et al. 2014 (22)	Investigate prognostic and predictive value of immunoscore in advanced melanoma patients treated with ipilimumab	190 FFPE metastatic samples from melanoma patients treated with ipilimumab	IHC expression of CD3, CD8, CD20 and FOXP3 on serial tissue sections	No relationship between CD3, CD8, CD20, CD163, FoxP3 both intratumoral (CT) and peritumoral (IM) with response/benefit; Only a trend for the CD163 positive PD-L1 positive population (p = 0.07)
Capone et al. 2014 (23)	Potential prognostic value of CD3, CD8, CD20, and FOXP3 as an ‘Immunoscore’ for melanoma	150 lymph nodes from 34 melanoma patients	IHC expression of CD3, CD8, CD20 and FOXP3 on serial tissue sections	Significant higher ratio of peri/intra tumoral CD3 and CD8 in patients without recurrence
Tumeh et al. 2014 (24)	Investigate adaptive immune resistance as predictor of response to anti-PD-1 therapy	Discovery cohort of 46 patients with FFPE material treated with anti-PD1 monotheray; Validation cohort of 15 patients	multiplex IF triple stainings, including S100, CD8, CD4, CD80, Ki67, pSTAT1, PD-1 and PD-L1	Predictive model for response to therapy based on CD8 expression at the invasive margin (after multivariate analysis)
Xu et al. 2017 (25)	Technique for measuring melanoma DoI in microscopic images digitized from MART1 (i.e., meleanoma-associated antigen recognized by T cells) stained skin histopathological sections	29 histopathological melanoma images (1 training, 28 validation images)	Four modules technique, including robust Bayesian based method for skin granular detection and multiresolution method using Hausdorff distance to measure melanoma invasion depth.	Superior performance in measuring the melanoma DoI of proposed multi-resolution approach compared to two closely related techniques.
Fertig et al. 2017 (26)	Compare concordance in differentiating spongiotic dermatitis (SD) and mycosis fungoides (MF) between digital whole-slide imaging (WSI) and traditional microscopy (TM )	20 cases of subacute SD and 20 cases of MF	WSI versus TM	Similar inter- and intraobserver discordance between WSI and TM
Kent et al. 2017 (27)	Compare accuracy/ reproducibility of pathologist in diagnosing dermatopathology cases between digital whole-slide imaging (WSI) and traditional microscopy (TM )	499 dermatopathology cases representing spectrum of diagnoses seen in the laboratory	WSI versus TM	Accuracy and reproducibility similar for WSI/TM
Xu et al. 2018 (28)	computer-aided technique for automated analysis and classification of melanocytic tumor on skin whole slide biopsy images.	66 H&E stained skin WSIs including 17 normal skin tissues, 17 nevi and 32 melanomas	multi-class support vector machine (mSVM) with extracted epidermis and dermis features	More than 95% accuracy for classifying a melanocytic image into different categories such as melanoma, nevus or normal tissue
Edwards et al. 2018 (29)	Prognostic value of tumor-resident CD8+ T cells in metastatic melanoma patients prior to immunotherapy and in patients undergoing anti–PD-1 immunotherapy	52 melanoma patients	multiplex IF using OPAL (CD8, CD103, SOX10, PD-1) & FACS	Increased numbers of CD69+CD103+ tumor-resident CD8+ T cells were associated with improved melanoma-specific survival in immunotherapy-naïve melanoma patients.
Halse et al. 2018 (30)	Prospective study explored the heterogeneous nature of metastatic melanoma using Multiplex immunohistochemistry (IHC) and flow cytometry (FACS)	FFPE from 21 melanoma patients	FACS & multiplex IF using OPAL (CD4, CD3, CD8, FOXP3, PD-L1, SOX10, CD20, CD68 and CD11c)	Model to define metastatic melanoma immune context into four categories using the presence or absence of PDL1+ melanoma cells and/or macrophages, combined with the presence or absence of IT CD8+ T cells
Onega et al. 2018 (31)	Compare accuracy/reproducibility of pathologist in diagnosing melanocytic lesions between digital whole-slide imaging (WSI) and traditional microscopy (TM )	180 skin biopsy cases including 90 invasive melanoma	WSI versus TM	Accuracy and reproducibility similar for WSI/TM
Thrane et al. 2018 (32)	Optimize and apply spatial transcriptomics (ST) technology for the in situ and quantitative detection of gene expression in stage III melanoma lymph node metastases	4 lymph node melanoma metastases	Spatial Transcriptomics AB	A detailed landscape of melanoma metastases was revealed by applying the ST technology to generate gene expression profiles, not evident through morphologic annotation
Johnson et al. 2018 (33)	Quantify immunosupression mechanisms within the tumor microenvironment by multiparameter algorithms to identify strong predictors of anti-PD1 response	Discovery cohort of 24 melanoma patients with FFPE material; Validation cohort of 142 melanoma patients with FFPE material	multiplex IF using OPAL (PD-1 & PD-L1, HLA-DR & IDO-1 and CD11b & S100); Analysis using AQUAnalysis ™	Patients with high PD-1/PD-L1 and/or IDO-1/HLA-DR more likely to respond (P = .0096) and have significantly improved progression free survival (hazard ratio [HR] = 0.36; P = .0004) and overall survival (HR = 0.39; P = .0011)
Alheejawi et al. 2019 (34)	Automatic measurement of proliferation index in Ki-67 stained biopsy image using deep learning algorithm	9 melanoma WSI	Convolutional neural network using SegNet architecture to segment and classify the Ki-67 stained image into three classes (i.e., background, active and passive nuclei	Robust segmentation/nuclei classification with average error rate less than 0.7%
Alheejawi et al. 2019 (35)	Computer Aided Diagnosis (CAD) method to segment the lymph nodes and melanoma regions in a biopsy image and measure the proliferation index	39 WSIs include 9 H&E, 9 MART-1, 9 KI-67, 5 CD-45, and 7 S-100 images	Local frequency features and SVM classifier for lymph node segmentation & Thresholding and SVM classification to determine active/passive nuclei	Segmentation of lymph nodes with more than 90% accuracy & proliferation index calculation with average error rate of less than 1.5%
Fu et al. 2019 (36)	systematic review of articles about the prognostic roles of TIL responses and CD3+, CD4+, CD8+, FOXP3+, and CD20+ TIL subsets in the prognosis of melanoma	41 studies included in final analysis	Systematic review & meta-analysis	Favorable prognostic role of CD3+, CD4+, CD8+, FOXP3+ and CD20+ TILs in melanoma
Wong et al. 2019 (37)	Are pretreatment tumor-infiltrating lymphocyte (TIL) profiles associated with response?	Study cohort of 94 anti-PD-1 treated melanoma patients; Historical cohort 100 untreated melanoma	5-plex IF using OPAL (including CD4, CD8, CD20, Ki67, GZMB)	Pretreatment lymphocytic infiltration is associated with anti–PD-1 response in metastatic melanoma
Robinson et al. 2019 (38)	Deep Neural Network (DNN) for quantitative prediction of melanoma recurrence from a H&E stained tissue	Training set of 75 melanoma patients; Validation cohort of 115 melanoma patients	Deep neural net (DNN) architecture consisting of convolutional and recurrent neural networks (CNN, RNN).	DNN recurrence prediction is independent prognostic factor in a multivariable Cox proportional hazard model
Wong et al. 2019 (39)	Test the hypothesis that CAF profiles in pretreatment tumor specimens are associated with response to anti-PD-1	Discovery cohort: 117 anti-PD1 treated melanoma patients; Control group: 194 melanoma patients	5-plex IF using OPAL (including Thy1, SMA, FAP, S100 and HMB45)	Pretreatment CAF profiles are associated with melanoma immunotherapy outcome
Gide et al. 2019 (40)	Examine the spatial distribution of immune and tumor cells to predict response to anti-PD-1-based therapies and patient outcomes	61 melanoma patients with FFPE material (27 monotherapy anti-PD1 treated; 34 combined anti-PD1 and anti-CTLA4)	multiplex IF using OPAL (PD-1, SOX10, PD-L1 and CD8)	Best model for 12-month progression-free survival for anti-PD-1 monotherapy included PD-L1+ cells within proximity to tumor cells and intratumoral CD8+ density (AUC = 0.80), and for combination therapy included CD8+ cells in proximity to tumor cells, intratumoral PD-L1+ density and LDH (AUC = 0.85)
Baltzarsen et al. 2020 (41)	Evaluate the diagnostic or prognostic marker of hTERT mRNA in melanoma	17 melanoma and 13 benign naevi	RNAscope	hTERT mRNA was more abundantly expressed in melanomas compared with benign naevi and correlated with the prognostic markers Breslow thickness and the Ki67 index
Cabrita et al. 2020 (42)	Investigate the role of B cells in antitumor responses in melanoma	177 melanoma patients	multiplex IF & Nanostring GeoMx Digital Spatial Profiler	Tertiary lymphoid structures have a key role in the immune microenvironment in melanoma, by conferring distinct T cell phenotypes & co-occurrence of tumour-associated CD8+ T cells and CD20+ B cells is associated with improved survival
Helmink et al. 2020 (43)	Investigate the role of B cells in antitumour responses in melanoma	Discovery cohort of 23 melanoma patients; Validation cohort of 18 melanoma patients	Gene expression profiling, multiplex IF using OPAL (CD20, CD21, CD4, CD8, FOXP3), Nanostring GeoMx Digital Spatial Profiler & CytOF	Potential role of B cells and tertiary lymphoid structures in the response to ICB treatment
Bosisio et al. 2020 (44)	Characterize the immune landscape in primary melanoma	29 primary cutaneous melanoma (23 non-brisk, 6 brisk)	multiplex IF using MILAN (39 plex), shotgun proteomics & qPCR	Brisk and non-brisk patterns are heterogeneous functional categories that can be further sub-classified into active, transitional or exhausted, and have an improved prognostic value when compared to that of the brisk classification
Ianni et al. 2020 (45)	deep learning system to classify digitized dermatopathology slides into 4 diagnostically-relevant classes (Basaloid, Squamous, Melanocytic and Other)	Training set of 5070 H&E stained skin biopsies; Validation set of 13 537 H&E stained skin biopsies	Deep learning system using a cascade of three independently-trained convolutional neural networks (CNNs)	Deep-learning-based confidence scoring classification system with accuracy of up to 98%
Chou et al. 2020 (46)	Compare the prognostic accuracy of an automated % TIL score using the NN192 algorithm to that of Clark’s grading	453 melanoma patients	TIL-quantifying neural network: NN192 algorithm	Automated % TIL scoring significantly differentiated survival using an estimated cutoff of 16.6% TIL, whereas TIL did not associate with RFS between groups (P > 0.05) when categorized as brisk, nonbrisk, or absent.
Kucharski et al. 2020 (47)	semi-supervised solution using convolutional autoencoders to to segment nests of melanocytes in histopathological images of H&E-stained skin specimens	Training set of 70 H&E stained WSIs of selected melanocytic lesions including 22 lentigo maligna, 20 junctional dysplastic nevi, 13 melanoma in situ and 15 superficial spreading melanoma (15); Validation set (of manually labeled ground truth images) of	Computer-vision based deep learning tool: Convolutional autoencoder neural network architecture with two semi-supervised training stages for the encoding and decoding parts	Segmentation of nests areas with Dice similarity coefficient 0.81, sensitivity 0.76, and specificity 0.94
Figueriredo et al. 2020 (48)	Investigate the mechanisms that supress tumor infiltrating lymphocyte in uveal melanoma	1 patient with uveal melanoma for Digital Spatial profiler,	Nanostring GeoMx Digital Spatial Profiler, CytOF and mRNA expression analysis	Loss of BAP1 expression is associated with an immunosuppressive microenvironment in uveal melanoma
Dikshit et al. 2020 (49)	Develop a novel workflow to combine the single molecule and single cell visualization capabilities of the RNAscope in situ hybridization (ISH) assay with the highly multiplexed spatial profiling capabilities of the GeoMx™ Digital Spatial Profiler (DSP) RNA assays	3 melanoma & 3 prostate tumors	RNAscope & Nanostring GeoMx Digital Spatial Profiler	Transcriptionally profiling of regions of high and low CTNNB1 expression within melanoma and prostate tumors and identify genes potentially regulated by the WNT- β-catenin pathway
Klein et al. 2021 (50)	Evaluate the predictive value of tumor infiltrating lymphocyte (TIL) clusters in primary MM and its association to molecular subtypes to predict response to CPI treatment.	H&E stained slides: Discovery cohort of 90 immune checkpoint therapy treated melanoma and a validation cohort of 351 patients from TGCA database	Deep-convolutional-neural network (U-Net) to detect viable tumor areas; following a quantitative TIL detection using a separate additional neural network	TIL clusters are associated with response to immunotherapy in BRAF V600E/K mutated MM.
Moore et al. 2021 (51)	Test whether automated digital (TIL) analysis (ADTA) improves accuracy of prediction of disease specific survival (DSS) based on current pathology standards	Training cohort of 80 melanoma patients, validation cohort of 145 melanoma patients	automated digital (TIL) analysis (ADTA) using a convolutional neural network (CNN)	After multivariable Cox proportional hazards analysis, ADTA contributed to DSS prediction (HR: 4.18, CI 1.51–11.58, p = 0.006).
Martinez-Morilla et al. 2021 (52)	Characterize the tumor microenvironment of patients with metastatic melanoma to find indicative factors of treatment response	Not reported	Imaging Mass Cytometry (IMC) (25 markers)	Identification of a series of potentially indicative biomarkers for immunotherapy in metastatic melanoma, including B2M.

### The New Morphological Evaluation: AI-Based

Historically, the role of the dermatopathologist in malignant melanoma concerned mainly 3 aspects: (i) find the right histopathological diagnosis of pigmented lesions; (ii) define the pathological staging for the primary malignant melanoma on the basis of Breslow thickness and ulceration; and (iii) list all the other relevant prognostic parameters not included in the staging process such as regression, inflammatory infiltrate, microsatellites, etc. This evaluation has always been done using a simple hematoxylin-eosin (HE) staining and a visual interpretation of the morphometric features of the tissue by the pathologist. The first task listed above is definitely the most challenging and still impossible to be performed by the machine autonomously. For the last two monotonous tasks, instead, the pathologist can be more effectively assisted by digital pathology where these parameters can be objectively quantified by the computer on digitized whole-slide images leaving more time to the pathologist for the diagnostic process.

**Figure 1:**
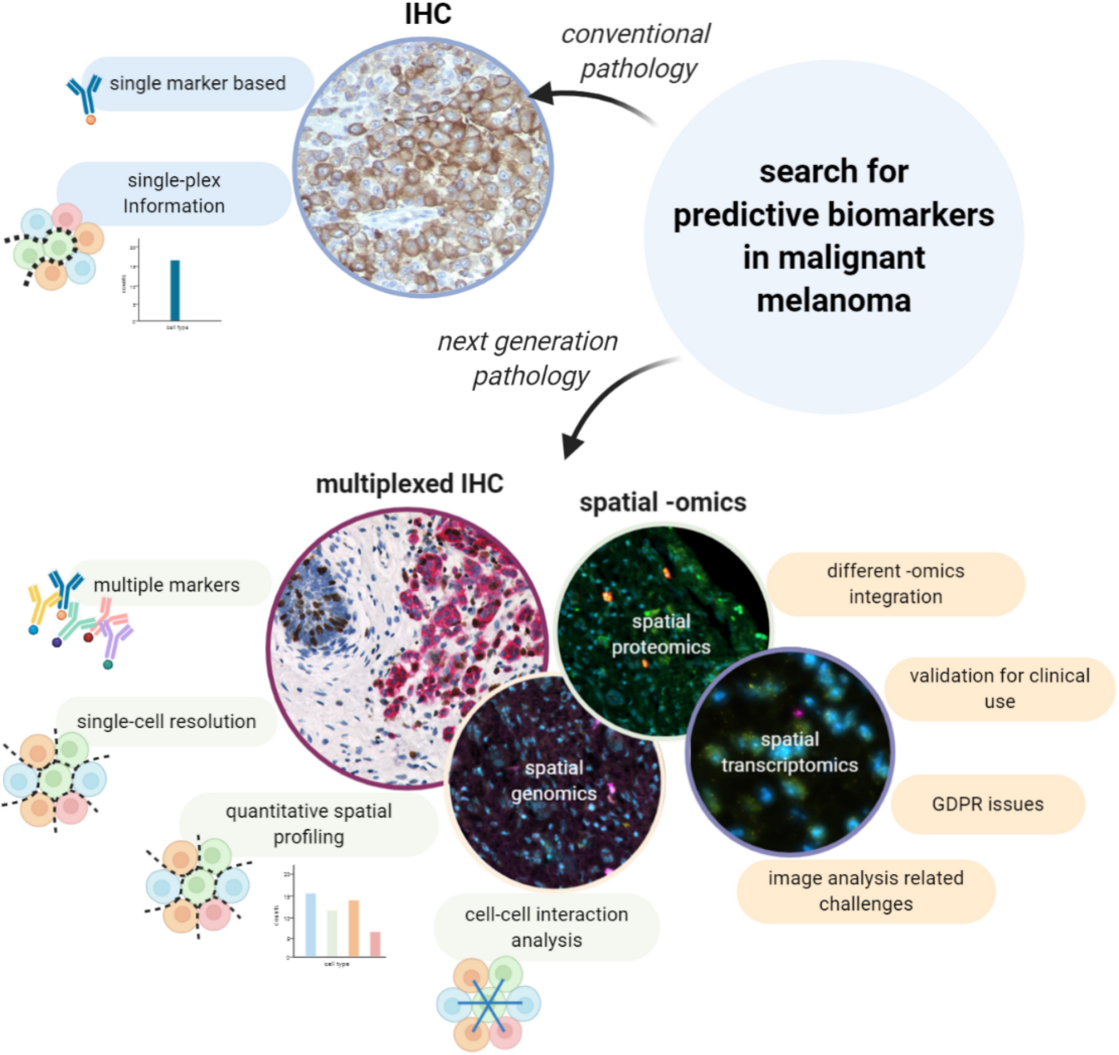
Digital Pathology and AI for a new morphological evaluation. The limitations related to the visual inspection-based diagnosis made by the pathologist on HE stained samples can be overcome with digital pathology and the support of AI and image analysis. Thanks to such computational tools it is possible to achieve more accurate diagnoses, based on a quantitative and more detailed analysis rather than a qualitative assessment, to support pathologists in their work, to automate time-consuming and repetitive tasks and to also improve the organization and the way cases are stored.

First of all, finding the right histopathological diagnosis of pigmented lesions is known to be one of the most challenging tasks in pathology, requiring extended training and expertise. This is further highlighted by the fact that there can be a high degree of discordance when the same lesion gets evaluated by different pathologists (53). Even though discordance is still present among the more experienced dermatopathologists (54), experience and specific training in dermatopathology do improve the diagnosis of difficult cases (55). In fact, digital pathology can be used to virtually share slides between peripheral hospitals and reference centers, facilitating the process of second opinion and expert review. As such, both AI and digital pathology can provide a more standardized level of diagnostic accuracy, ensuring patients get access to the most reliable diagnostic assessments. Digitized whole scan images of a histological slide have been found to have similar effectiveness, both in terms of accuracy and diagnostic workflow, to traditional microscopy for the evaluation melanocytic lesions (26, 27, 56). Moreover, artificial intelligence can also bring its experience, namely its machine learning training, to the side of less experienced pathologists to assist them with more complex diagnostics. In this direction, even before the introduction of machine learning, feature extraction-based algorithms had already proven to be efficient to distinguish melanocytic lesions with an accuracy of 95% (28). Even more recently, a first machine learning algorithm was developed to evaluate the degree of uniformity and symmetry of melanocytic nests as a first step to discriminate between benign and malignant lesions (47). Nevertheless, it is very unlikely that the application of digital pathology and AI will replace the pathologist in the diagnostic process, especially for melanocytic lesions. Since the use of deep learning allows the mining of complex morphometric features that go beyond mere visual identification, these can be applied in the form of an augmented reality rather than of an autonomously working AI, in order to suggest elements in favor and against the diagnosis of melanoma that will necessarily need to be reviewed by the pathologist itself. The augmented reality will bring to the attention of the pathologist features that should not be missed, helping him to recognize the trivial case (all the features pointing in one direction) from the more complex one (more contrasting/ambiguous features), speeding up the work of the pathologist, thanks to a triage process but not substituting him in making the definitive diagnosis. Therefore, it is also more realistic that the role of digital pathology and machine learning will be assisting the general pathologists with less experience in melanocytic lesions rather than the experienced dermatopathologist (**Figure 1**).

Interestingly, artificial intelligence could also be used to organize collections of digitized tissue slides by image similarity, and, as such, go far beyond the use of mere text-based searches. This can have various applications: (i) matching new cases to archived morphologically similar cases to propose a putative diagnosis and potentially improve the diagnostic accuracy; (ii) groups of similar images can more efficiently be retrieved from the archives for training purposes, not only to develop new or improved algorithms, but also for pathologists-in-training (57).

Software packages that are able to apply automated measurements, can also make diagnostics more efficient by automatically retrieving the required parameters and adding them to clinical reports. One of the first studies to use deep learning in histopathology allowed to recognize and count mitotic cells in breast cancer with higher accuracy compared to manual assessment (58). As manual counting mitotic nuclei is a highly time-consuming tasks, it could be easily replaced by AI in melanoma reporting as well. Other practical examples involve the measurement of the Breslow thickness (25), the evaluation of the proliferation index or the detection of lymph node metastasis (34, 35), for which deep learning algorithms are already available. In addition to this, deep learning has been proven useful as an alternative way to the most traditional pathological report to predict the risk of melanoma recurrence, on the basis of features extracted from HE images (38). Moreover, image analysis and machine learning were also applied to quantify tumor infiltrating lymphocytes (TILs) on HE, revealing to be a better tool than the actual semiquantitative classification in brisk, non-brisk and absent to estimate survival for melanoma patients (46, 50, 51) and to be associated with response to checkpoint inhibitors in BRAF V600E/K mutated malignant melanomas (50)

Finally, on top of assisting the pathologist with the diagnostic process and the definition of the prognosis, there are additional advantages to the introduction of digital pathology (**Figure 1**). The number of cases in dermatopathology has been rising over the last decade, and as such also the workload of the dermatopathologists (59). Most of these lesions are benign and easy to recognize, yet require dedicated time for evaluation. This reduces the available time for the more challenging/difficult cases. Software packages have recently entered the market that can assign a “class” to a skin lesion (e.g. epithelial vs melanocytic), detect easy, benign lesions, that can be prioritized and quickly diagnosed, and assign a particular flag to cases recognized as “complex”, onto which the pathologist can focus longer (45). In this way, artificial intelligence can help to optimize the flow of the daily work of the pathologist and improve the robustness and efficiency to come to a proper diagnosis.

### Beyond Morphology: The Spatial Omics

**Figure 2:**
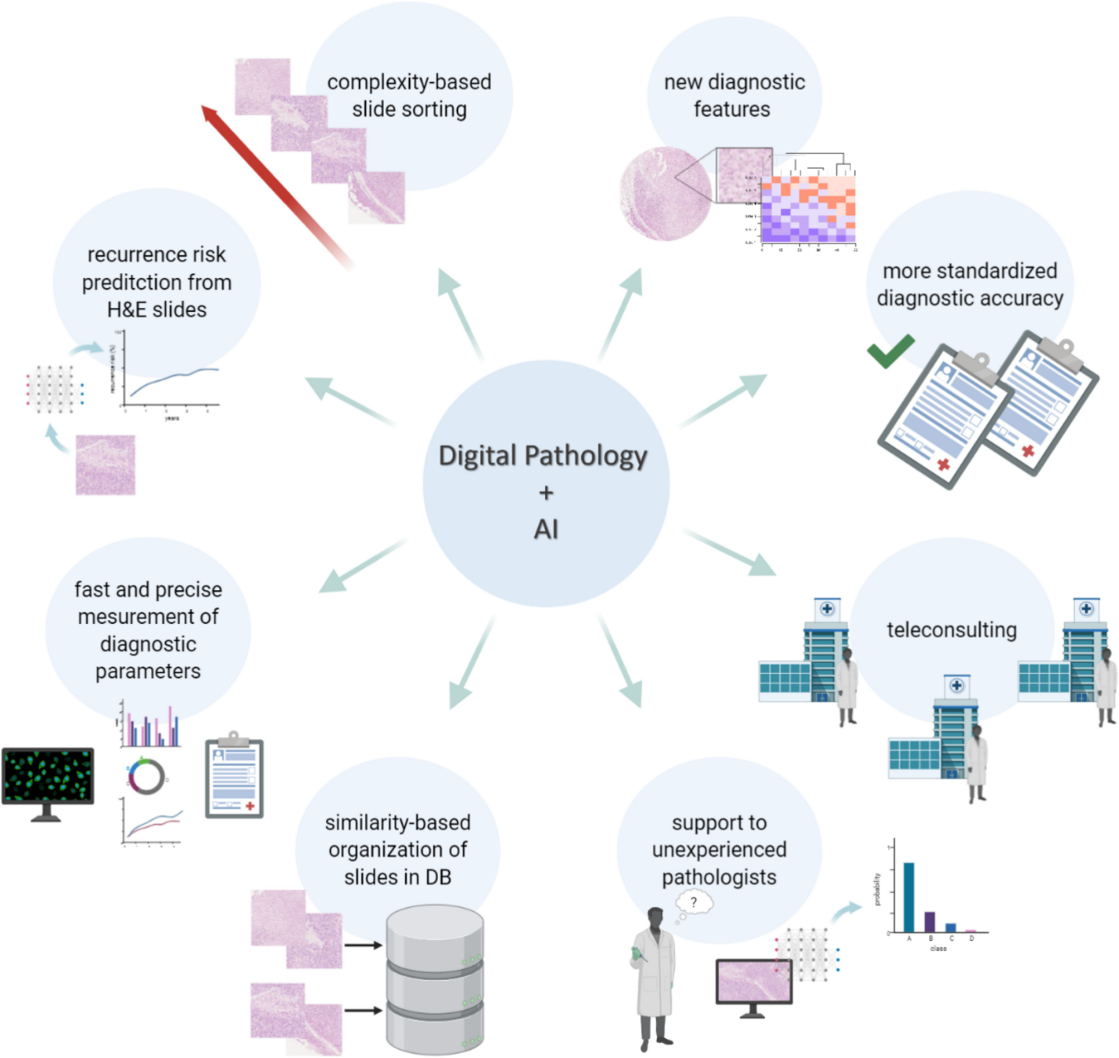
Searching for predictive biomarkers in malignant melanoma with spatial multiplexing techniques: advantages and challenges. Predictive evaluation of malignant melanoma is needed for a more personalized treatment plan, but predictive biomarkers must still be identified. Conventional IHC is a single-plex based method which does not provide information at single-cell level. On the other hand, multiplexed IHC and spatial -omics methods make it possible to extract information from multiple markers at single-cell resolution and to investigate cell-cell interactions. However, despite the great advantages, those techniques have not yet been validated in clinics and it is currently not possible to integrate the information from different -omics on the same section at single-cell level. Moreover, those methods are strictly dependent on computational techniques for the downstream analysis, hence they carry all the challenges related to image analysis.

As stated higher, the evolving treatment landscape in malignant melanoma has resulted in an increased demand for more and better predictive evaluations on top of the already available prognostic ones. The combination of both is a prerequisite to move toward personalized approaches in which treatments are matched to the right patients. Within metastatic melanoma, the use of checkpoint inhibitor therapy has revolutionized the outcome for patients with an objective response rate between 33.7-45%. Interestingly, the clinical efficacy of anti-PD1 antibodies as monotherapy (15, 16, 60), was slightly improved when combined with anti-CTLA-4 antibodies (up to 58%) (60, 61), but at the cost of higher toxicity rates. To avoid the biological, ethical and economical costs of administering non-effective treatments to patients, we will need to find predictive biomarkers that can guide clinicians to make informed decisions. In this light, several biomarkers have been described, such as a minimal expression of PD-L1 by conventional IHC (62), a minimal level of tumor mutational burden (TMB) (63), and gene expression profiling (GEP) using the IPRES or IMPRES signatures (64, 65), but none of these have provided the required sensitivity and/or specificity to be implemented in the clinic. This could be due to the limited amounts of information on the tumor and its microenvironment that are gathered by these assays, and which turned out to be insufficient to efficiently predict response to therapy. Indeed, understanding the conditions in which the immune system can be reinvigorated by ICB turns out to be complex and requires the integration of multiple parameters and features. Next-generation pathology using spatially resolved single-cell assessments of a tissue has the potential to shed more light on the complex role of the TME in a patient response to therapy, as it integrates functional information of each individual cell while adding information about their spatial context (**Figure 2**) and as such the interactions between different cell types.

As anticipated in the introduction, conventional IHC cannot provide a multiparametric in-depth characterization of the tissue at single cell level. To overcome the limitations of conventional IHC, multiple approaches have been tested. A first example involves the use of virtual multiplexing which vertically aligns digital images from serial sections. Virtual multiplexing has been made (commercially) available by VisioPharm and HistogGeneX (66) among others. An example is the Tissuealign™ analysis module from VisioPharm that has been validated for *in vitro* diagnostic use (CE-IVD) in Europe in combination with the CE IVD APPs from VisioPharm (67, 68). Nevertheless, vertical registration still does not allow detailed single-cell phenotyping which requires insights in the co-expression of different markers in exactly the same cell. In addition, to identify all the inflammatory subpopulations that are present in a histological sample, the evaluation of more than 20 markers is needed, ideally on the same tissue section (“high-plexing”). Nowadays, several methods for tissue multiplexing are available (69) and any technique representing a surrogate to investigate co-expression of markers at single cell level should be replaced by multiplexed IHC. First investigated in the context of colorectal cancer (70), the implementation of the concept of an ‘Immunoscore’ or immunoprofiling into a renewed cancer staging system incorporating the effects of the host immune response based on the numeration of specific lymphocyte populations alongside with the tumor cell-autonomous characteristics has been proven useful in the context of advanced melanoma as well (23). The colorectal Immunoscore, which involved a quantitative assessment of CD3^+^ and CD8^+^ T cells both at the invasive margin and bulk of the tumor, was already published in 2006 and encouraged the adoption of digital pathology tools for biomarker discovery (70, 71). Specific for melanoma, the definition of a comparable Immunoscore seems to be a more difficult challenge (72). In many patients, metastatic lymph nodes are the only available tissue samples and concerns are raised about the applicability of an Immunoscore in lymph nodes because they are constitutively rich in CD3 and CD20 lymphocytes. In a first effort, an Immunoscore constructed based on the expression of CD8, CD3, CD20 and FOXP3, was applied on a small cohort of stage III melanoma patients showing significant differences in the peri/intratumoral ratio for both CD3 and CD8, with the ratio being higher in patients without recurrence compared to patients with melanoma recurrence, with similar trends for both FOXP3 and CD20 were observed (71). In a more recently published systematic review, a favorable prognostic role of the CD3^+^, CD4^+^, CD8^+^, FOXP3^+^, and CD20^+^ TILs on the overall survival of melanoma patients was confirmed. In addition, in a subgroup analysis, brisk TILs were associated with overall survival, recurrence-free survival, and melanoma-specific survival (36). Likewise, the predictive performance of an alternative Immunoscore, using a digital image analysis application to characterize immune infiltrate expression of CD3, CD8, CD20, FOXP3 and CD163 and of PD-L1, was tested in a metastatic melanoma cohort of patients treated with Ipilimumab in the MISIPI trial (22, 72). Unfortunately, this trial was unable to confirm the relationship between intra/peritumoral expression of CD3, CD8, CD20, CD163, FOXP3 and a response/benefit to therapy, apart from a trend for the CD163-PD-L1 double positive population (22). Another study, using a low-plex with only 6 markers found instead that the quantity but not the activation of CD8+ TILs was associated with anti-PD-1 response in metastatic melanoma (37). In an attempt to categorize the intrinsic heterogeneous nature of metastatic melanoma, Halse and colleagues used multiplex immunohistochemistry to provide a model which defines the immune context into four categories, using the presence or absence of PD-L1^+^ melanoma cells and/or macrophages, and their location within or around the tumor, combined with the presence or absence of intratumoral CD8^+^ T cells. This model values the melanoma TME as a spectrum between tumor escape and tumor (immune) control within the space of a tissue (30), encouraging others to investigate the spatial distribution of both immune and tumoral cells when interpreting the response to immunotherapy. Confirming the latter, whereas no association with response or survival could be observed in the expression of individual biomarkers (PD-1, PD-L1, IDO-1, HLA-DR), a spatially-resolved low-plex PD-1/PD-L1 interaction score and/or IDO-1/HLA-DR co-expression was strongly associated with an anti–PD-1 response, highlighting the importance of quantitative spatial profiling for multiple features (33). Furthermore, Gide and colleagues examined the spatial distribution of immune and tumor cells using a 5-plex immunofluorescence approach in samples of patients prior to a treatment with either anti-PD-1 monotherapy or a combination of anti-CTLA-4 and anti-PD-1. In a multivariate analysis, the best predictor for a 12-month progression-free survival upon anti-PD-1 monotherapy involved the quantification of the proximity of PD-L1^+^ immune cells to tumor cells and the density of intratumoral CD8^+^ T-cell, as such achieving an AUC of 0.80. For the combination therapy, the authors identified that a correlation with the proximity of CD8^+^ T-cells to tumor cells, the density of intratumoral PD-L1^+^ cells and LDH expression (AUC = 0.85) to response to therapy (40). Similarly, others have shown that pre-treatment samples obtained from responders to anti-PD1 therapy showed that increased amounts of CD8^-^, PD-1^-^ and PD-L1^+^ cells resided at the invasive tumor margins and within the tumor, with close proximity the ligands PD-1 and PD-L1 (24). In addition, the use of multiplexed immunofluorescence highlighted a potential predictive role for specific cancer-associated fibroblasts (39) and CD103^+^ tumor-resident CD8^+^ T cells (29) in melanoma patients treated with anti-PD-1 therapy. Also, the potential contributing role of tumor-associated B-cells has been studied using multiplexed immunofluorescence, showing an association between co-occurrence of tumor associated CD8^+^ T cells and CD20^+^ B cells with improved survival, while revealing the formation of tertiary lymphoid structures (TLS) in these CD8^+^CD20^+^ tumors (42) and their potential role in response to immunotherapy (43). Overall, according to a recently published meta-analyses, the extended information that could be extracted using multiplexed IHC/IF appears to be associated with improved relative diagnostic accuracy in predicting clinical response to anti-PD-1 therapy over the other previously mentioned biomarkers (PD-L1 conventional IHC, TMB, GEP) (73).

Strikingly, most of the research trying to map the melanoma TME were done using “low-plex” methods (less than 10 markers on the same section), and yet the majority of them added interesting insights into the anti-melanoma immune response and response to immunotherapy. The reason why even a low-plex approach can be more insightful compared to other molecular methods (e.g. NGS analysis) which often cover even more parameters, could be related to the insights gained within the spatial component. Indeed, the spatial dimension (i.e. understanding the exact position of each cell type within a tissue) could be considered as a biomarker itself. Available analysis methods are now able to generate cell density metrics for specific tissue regions, assess the distance between various cell types, among many more. Such higher-order insights grant the possibility to go beyond mere cytometric analysis of the tissue (i.e. the overall cellular composition of a tissue) and investigate “cellular sociology” in order to make assumptions about their interactions in particular niches. Moreover, already in the early days of single-cell genomics, it was clear that the success of the different single-cell technologies would depend, in part, on the extent to which researchers preserve the states of cells and the original composition of a tissue (74). After all, most of the initial single-cell methods required cells to be dissociated from the tissue, thereby losing all spatial information while potentially affecting the original cell states. To deal with this flaw, the most recent single-cell methods aim at preserving the cells in their original context and state.

Within the available spatial omics methods, we can distinguish spatial proteomics, transcriptomics and genomics (**Figure 2**). Most of the spatial proteomics techniques are antibody based methods that achieve their plexability from either multi-spectral imaging and/or iterative imaging of successive antibody staining cycles combined with fluorophore bleaching/inactivation/cleaving or antibody stripping (75–80). Similarly, spatial transcriptomics enable the in-situ visualization of RNA transcripts within the tissue either by measuring pre-determined targets or even global expression data (81–86). A detailed description and comparison of the different available techniques for spatial -omics, goes beyond the scope of this review.

Several of the abovementioned technologies have been applied to melanoma in an attempt to improve prognostic and predictive performance of potential biomarkers, or as a discovery tool to unravel mechanistic insights in the TME. Accordingly, our group previously used MILAN – an imaging/antibody-based single-cell proteomics method - to functionally study tissue architecture, thereby redefining the TIL infiltrate in primary melanoma into a functional classification with an improved prognostic value as compared to the dogmatic morphological classification (44) and described a higher level of interaction between melanoma cells with active CD8^+^ and CD4^+^ T cells in patients responding to anti-PD-1 as compared to the non-responding patients (87). Others have used imaging mass cytometry (IMC) with a 25-antibody panel to identify tumor and immune cell markers in melanoma patients treated with immune checkpoint blockers, revealing significant associations of MHC-I, CSF1R, IRF1, LAG-3, PD-1, MHC-II and beta2-microglobulin expression in tumor tissue with progression-free survival, whereas high levels of TIM-3 and PD-L2 in the stroma also predicted response to immunotherapy (52). Spatial transcriptomics have been used to visualize the distribution of mRNA within the melanoma TME, revealing among others the complex transcriptional spatial landscape and genetic heterogeneity in stage III cutaneous melanoma (32).

### The Integration of Multi-Omics Within the Tissue

Most of the methods for spatial omics are limited by their ability to examine only one type of analyte (protein or nucleic acids). One step closer toward a complete understanding of the TME of melanoma and the drivers of response to immunotherapy will require the integration of information retrieved from several -omics approaches, each providing complementary information. The integration of information from different omics technologies and particularly those integrating information from the same section at single-cell level while preserving the spatial context represents the ultimate goal for next-generation pathology. In spite of major progress in the development of methodologies that simultaneously extract various features from the same cell, a genuine, spatially integrated multi-omics approach, enabling the simultaneous analysis of proteome, transcriptome, genome- and epigenome within the spatial coordinates is still not available. Therefore, while the development of such combinatory technologies is ongoing, other approaches are being evaluated, including the computational integration of spatially resolved assays (which typically rely on a predefined, but limited set of features), and an unbiased method, such as single-cell RNA-sequencing (scRNAseq) which requires cells to be removed from the tissue. Another simplified approach consists of performing various spatially resolved omics analyses on serial sections, and integrating the findings in a comprehensive framework. As discussed earlier, the use of multiplex immunofluorescence confirmed the importance of tumor-associated B cells and TLS in metastatic melanoma (42, 43), a finding that was further corroborated by spatial transcriptomics, indicating that T cells in TLS-negative tumors had a dysfunctional molecular phenotype (42) whereas TLS-positive tumors are associated with markers of T cell activation and B cell proliferation (43). Alternatively, combining multiple omics on serial sections can be integrated at the tissue level by making use of vertical registration. For example, digitally superimposing a melan-A classical IHC on 1 section and RNAscope probing for hTERT on the adjacent section, allowed these researchers to show a higher expression of hTERT mRNA in melanoma as compared to benign naevi (41). Yet another approach is to laser-microdissect regions from a tissue for gene-expression analysis, while being guided by classical IHC staining on the adjacent section. Such approach has been used to compare expression profiles of IHC-positive and IHC-negative areas, thereby improving cell-type purity in the different samples as compared to classical tissue-based transcriptomic data (21). With the development of Spatially-resolved Transcriptomics *via* Epitope Anchoring (SvEA) in pivotal work done by Govek and colleagues, this has recently been made possible (88). In this approach, the transcriptomic data acquired *via* CITE-seq (89) can be mapped to spatially resolved CODEX mIHC data (77), while retaining the single cell spatial resolution by making use of measurements of the same antigens in both methods (88). In spite of the obvious translational potential of this novel approach, it has not yet been applied within the melanoma field. More recently, the GeoMx^®^ DSP (Digital Spatial Profiling) platform has been made commercially available (90). This platform allows protein or RNA quantification within user-defined regions-of-interest (ROI), with the possibility of single-cell resolution ROI selection (The UV laser can be focused as narrow as 10 µm in diameter). This ROI selection is achieved by combining regular, low-plex IF staining together with dozens of primary antibodies or mRNA hybridization probes each covalently attached to indexing oligonucleotides that can be collected for quantification using a UV-photo cleavable linker (91). Using this method, Dikshit and colleagues recently transcriptionally profiled regions with high and low Beta-catenin expression in melanoma, showing a significant correlation with several immune-regulatory targets such as CTLA-4 and PD-1 (49). Similarly, the method was used to show a specific expression profile in fibrotic areas with high macrophage and T cell infiltration in BAP-1 negative uveal melanoma, suggestive of T-cell exhaustion other than PD-1/CTLA-4 engagement, as well as mechanisms of immune exclusion, supporting the clinical observation of immunotherapeutic failure in this subgroup and the need for development of specific treatment approaches (48). Two other studies have used DSP to characterize the tumor expression profile of melanoma patients treated with immune checkpoint blockade in a neoadjuvant setting, showing that baseline immune infiltration was correlated with response to treatment (92, 93). Although offered as an easy applicable method, a detailed understanding of its composition, function and chemistry is advisable to guide experimental design and data interpretation (94).

Finally, AI will have a predominant role in the integration of all the different data types. In the previous section we have discussed how AI has already outperformed pathologists on evaluating morphological features such as mitotic counts (58), Breslow thickness (25) or at detecting lymph node metastases (34, 35) - routine tasks that could significantly enhance the throughput and efficiency of pathologists while ensuring sufficient can be spent on complex cases. However, the strongest ability of AI, and in particular deep learning, is to identify unknown patterns or features that are too complex for pathologists to merely assess by visual eyeballing but which could be of important diagnostic, prognostic, or predictive relevance. This is already true in the case of morphometric features (95). In the case of spatially-resolved, single-cell multi-omics data, the feature space is still significantly larger and the number of hidden associations that could be used as biomarkers is virtually endless. For their translatability to clinical practice however, these complex features require first to be re-engineered to simpler biomarkers or simpler algorithms that identify the specific discriminant features which might be more easily accepted by clinicians (96).

## Discussion: Next-Generation Pathology and Its Challenges

Even though next-generation pathology is becoming gradually more prominent and qualifies as a necessity in research, very few of the previously discussed developments have yet been validated for clinical use. With the increasing importance of understanding the immune contexture and the possible development of panels of prognostic/predictive biomarkers across multiple diseases, we foresee that the implementation of next-generation pathology in clinical practice will be mandatory. However, there are still a lot of hurdles and challenges that need to be overcome before multiplexed IHC and digital pathology will be implementable in clinical practice.

The main difficulty of implementing multiplexed IHC is to overcome the common thinking that it is based on the repetition of multiple conventional IHC assays. Indeed, multiplexed IHC is a complex process and there are various challenges that need to be considered. The first challenge is about choosing the most appropriate method. This choice should consider several factors. First of all, the type of samples to be analyzed: some methods require FFPE materials while others can be performed on frozen samples. In a standard clinical pathology lab, FFPE remains the method of choice to preserve tissue specimen, even though multiple methods (mainly multi-omics) require the availability of frozen materials. The second choice will be to related to the actual staining procedure: this can be achieved either by (i) a cyclic method, in which slides are stained multiple times with low-plex antibody cocktails while between every cycle the signal is removed *via* antibody stripping or bleaching of the fluorophore; or (ii) an all-at-once acquisition, where a cocktail containing all the antibodies of choice are applied on the tissue section in a single step. In the first case the acquisition will be slower, while in the second case the increased speed of acquisition will increase technology costs. For instance, several methods require modified/engineered/conjugated antibodies (e.g. with nucleotide barcodes or metal ions) that are more expensive than conventional clones typically used in routine across clinical pathology labs. On top of this, the instruments that are used to detect the signals are often a factor 2-3 more expensive than conventional autostainers typically used for classical IHC. These machines are generally closed systems that have the advantage to be completely automated, requiring less work by the lab technician being therefore less prone to errors. Nevertheless, these methods may be limited to the acquisition of regions of interest rather than whole slides; depending on the number of antibodies to be detected the acquisition time could require even hours per square mm. A final parameter to consider on the wet-lab part is the number of samples that should be analyzed simultaneously: while some methods allow the analysis of a single slide at the time, others are compatible with batch processing.

The second big challenge of implementing multiplexed IHC in hospital routine relates to data analysis. At the moment, most of the wet-lab methods are not paired with a system to analyze the data. Importantly, multiplexed IHC can no longer be evaluated through mere visual inspection (as opposed to conventional IHC where it is common practice), but requires specific methods for quantitative, spatially resolved analysis. Therefore, until *ad hoc* software packages will be introduced for specific predictive/prognostic analyses, experts in image analysis and bioinformaticians will remain required for the downstream analysis. In addition, a simple panel of 10 markers will generate 100 digital images when analyzing 10 samples, an amount that will steadily increase when increasing numbers of markers and samples are processed. This is where the hurdles to implement multiplex IHC converge with those of implementing digital pathology.

As described above, digital pathology bears the potential to revolutionize dermatology and dermatopathology. To achieve its implementation, though, multiple challenges have to be overcome, and typically involve hurdles that are cultural, involve validation, available infrastructure and GDPR-related issues. From a cultural point of view, in spite of the advantages listed in the first chapter of this review, pathologists still show some reservations about the use of digital slides for diagnosis, mainly regarding the time needed to evaluate whole digital slides during routine work, with a preference to reserve the digital format for teaching, second opinions and dissemination purposes (31). Second, appropriate validation remains an absolute requirement for any new technique that get implemented for diagnostic purposes. Such validation does not solely happen at the level of a company trying to sell a diagnostic tool, but can also happen directly at a local level, for instance in a pathology department that is willing to introduce a new technique in its workflow. Validation itself will mainly require side-by-side comparisons of manually and digitally interpreted tissue slides (97). Validation is also required for deep learning algorithms, which have the danger to be based on overfitted or miscorrelating data from training sets. In particular, on one side algorithms have to be strictly disease-specific and exclude any other disease that may be encountered during analysis (e.g., algorithms developed for the analysis of melanoma should recognize and revoke the analysis of any other skin tumor). On the other side, since they are extremely dependent on their training, it is important to be aware that if important differences are introduced in time that may artificially change the features of the prospectively collected diagnostic data set, high rates of misclassification can be registered (98). Next to these first two challenges, there are also important infrastructural challenges to be considered that can hamper the implementation of digitization in a clinical institution. A standard microscopy slide, such as the ones routinely used in pathology are typically 75 mm long, 26 mm wide, and approximately 1 mm thick. As the resolution and color depth of digital detectors improve, the size of images that capture these slides keeps on increasing. For a state-of-the-art acquisition instrument with a resolution of 0.44 micrometers/px (20X) and color depth of 16 bit (~65K gray levels), and assuming a 2-dimensional slide with standard dimensions, single images achieve a size of ~20 Gigabytes (Gb). Of course, depending on the size of the scanned area and the type of image compression, whole slide images can range between 0.5 and 4 Gb (96). This is translated to hundreds of Terabytes per year (or even Petabytes when considering a large hospital) that need to be properly acquired, stored, transferred, and processed. Designing and implementing a proper infrastructure for digital pathology that deals with all these tasks is not trivial and key for a successful digital transition. Regarding image acquisition, this is an easily solvable problem, since in the 20 years since the introduction of whole-slide imaging scanners, several of them have been marketed for clinical use in the European Union and in the US (99, 100). Image storage and transfer are instead critical steps and they may require important investments on the side of the institution. There exist different types of solutions for image storage, ranging from local ones such as Direct Attached Storage (DAS), network-based solutions such as Network Attached Storage (NAS), cloud-based solutions (such as Amazon’s S3 Glacier storage for example) or external services (regional supercomputer centers). The last two examples require sending data to third parties which could have GDPR issues (see below). In most cases, several of these solutions need to be simultaneously implemented to archive the data depending on different factors, including access frequency (hot/interactive *versus* cold/archival storage) or intended use (101). For image transfer, solutions where data is remotely stored or/and the images are remotely analyzed, the speed in which data is transferred (network bandwidth) becomes a critical factor. If we consider a 10 Gb image and a standard Local Area Network (LAN) with a bandwidth of 100Mbit/s, it will take ~15 minutes to transfer the file. Therefore it is crucial to guarantee an environment with sufficient bandwidth prior to taking the step to digital pathology. Finally, most of the digital image analysis algorithms currently used in clinical practice are limited to traditional image analysis and can be used on ordinary computers with Central Processing Units (CPUs) (96). Deep learning algorithms, on the other hand, are heavily dependent on processing acceleration units such as Graphical Processing Units (GPUs) (102). High-end GPUs are very expensive and therefore centers implementing deep learning in digital pathology might choose for a dedicated workstation/server or even to train/run their algorithms in the cloud or in external supercomputer centers. Lately, the development of Tensor Processing Units (TPUs) are allowing the training of deep neural networks (DNN) 15-30 times faster and 30-80 times more energy efficient than contemporary CPUs or GPUs (103). Additional infrastructural challenges to be considered for implementing deep learning in digital pathology include: the number of users of the dedicated computers, the flexibility of the system to implement new algorithms or variable case-loads, implementation/running cost of the facility, cyber-security, data maintenance, etc. (96). A practical example of the implementation process of a fully digital workflow at the University Medical Centre in Utrecht can be found in Stathonikos et al. (104).

The last challenge for digital pathology is correlated with the fact that digital images, as well as patient materials, are subject to the regulation on data protection and privacy in the European Union and the European Economic Area on the protection of natural persons with regard to the processing of personal data and on the free movement of such data (General Data Protection Regulation, GDPR) (105). With respect to digital pathology, it contains several basic principles that digital slides containing human samples must comply with. These include: Purpose specification, the valid legal basis for the collection of the data including the goal for which the data is being collected (106); anonymization or pseudo-anonymization, data is only anonymous when it is impossible to track it to natural persons while pseudo-anonymous data requires extra information to map it to natural persons (107, 108); Data minimization, the collected data should be limited to what is strictly necessary for the scope of the project (107) transparency, the registration of the study and the provision of the relevant information to the subject of the study; storage limitation, the collected data should only be kept as long as needed; and security, the stored data should be processed and stored in such way that it avoids or limits the potential for unlawful processing, accidental loss, destruction or damage (107). This includes technical measures such as badge and password-mediated access control, detailed logs monitoring every ongoing process on the system, data encryption, etc. If the data is stored on the cloud or in external sources, a legal contract needs to be written between the collector and the third party which needs to be checked by legal entities (109, 110).

To conclude, the highly requested demand for a better understanding of the TME in melanoma and its use to further improve the clinical response rates to immunotherapy, the fast-moving technological advancements in machine learning and the rise of spatial omics, have pushed dermatopathology into the digital era. Although the use of digital pathology has already proven to be insightful in melanoma, its exploitation to the full potential by combining spatially resolved single-cell data with artificial intelligence for clinical purposes, is still a rather future perspective. However, such an approach possesses the capability to overcome existing limitations and bring us one step closer to personalized medicine. Nonetheless, despite the many advantages, a lot of the imaging-based methods go along with substantial challenges that need to be addressed before its implementation in daily practice will be possible.

## Author Contributions

YH decided the contents, did the main literature research, wrote all the parts about the omics, reviewed the format and the structure of the paper. AA wrote the part on the dry lab challenges and reviewed the format and the structure of the paper. MA researched the topic on GDPR regulations and reviewed the paper. GM created the images and reviewed the paper. OB reviewed the paper from the oncologist point of view. FS reviewed the paper from the bioengineer point of view. FB decided the contents, structured the flow of the paper, gave the pathology insights, wrote the introduction, the part about AI beyond morphology and the part on the discussion about the wet lab challenges. All authors contributed to the article and approved the submitted version.

## Funding

AA was supported by the Leuven Kankerinstituut (LKI) and the Opening The Future (OTF) foundation. This work is also supported by the Kom op tegen kanker (KOTK) foundation.

## Conflict of Interest

The authors declare that the research was conducted in the absence of any commercial or financial relationships that could be construed as a potential conflict of interest.
